# Potential regulatory role of the m ^6^A-lncRNA axis in breast cancer: molecular mechanisms and therapeutic implications: Potential role of the m ^6^A-lncRNA axis in breast cancer

**DOI:** 10.3724/abbs.2025134

**Published:** 2025-07-28

**Authors:** Di Chen, Jinyan Wang, Xichun Hu, Shu Liu

**Affiliations:** 1 Department of Breast Surgery the Affiliated Hospital of Guizhou Medical University Guiyang 550004 China; 2 Department of Clinical Medicine Guizhou Medical University Guiyang 550004 China; 3 Department of Medical Oncology Fudan University Shanghai Cancer Center Shanghai 200032 China; 4 Department of Oncology Shanghai Medical College Fudan University Shanghai 200032 China

**Keywords:** breast cancer, m ^6^A, lncRNAs, clinical therapy

## Abstract

N ^6^-methyladenosine (m ^6^A) modification, the most prevalent internal modification in eukaryotic messenger RNAs (mRNAs), has emerged as a crucial regulator of various biological processes. This reversible epigenetic modification is dynamically regulated by methyltransferases (writers), demethylases (erasers), and m ^6^A-binding proteins (readers). Aberrant m ^6^A modification is associated with the initiation, progression, and metastasis of breast cancer, highlighting its potential as a therapeutic target. Long non-coding RNAs (lncRNAs), a class of non-protein-coding transcripts longer than 200 nucleotides, are also involved in breast cancer development through diverse mechanisms. Increasing evidence suggests a complex interplay between m ^6^A modifications and lncRNAs in breast cancer, with lncRNAs modulating m ^6^A regulators and m ^6^A-modified lncRNAs exerting functional effects. This review comprehensively summarizes the current understanding of the m ^6^A-lncRNA axis in breast cancer, including the molecular mechanisms underlying its interaction and its effects on breast cancer biological processes, such as proliferation, apoptosis, migration, invasion, and therapy resistance, and highlights the potential of this axis as a diagnostic and therapeutic biomarker. Additionally, we discuss the challenges and future directions in this rapidly evolving field, aiming to provide insights for the development of novel therapeutic strategies for breast cancer.

## Introduction

Breast cancer remains the most common malignancy in women worldwide, with 2.3 million new cases and 670,000 deaths reported in 2022, representing an annual increase of 1%–5% [ 1, 2] . Its molecular heterogeneity is classified into four major subtypes: luminal A, luminal B, HER2-enriched, and triple-negative breast cancer (TNBC), each presenting distinct therapeutic challenges. While advances in surgery, radiotherapy, chemotherapy, endocrine therapy, and targeted therapies have improved outcomes, more than 30% of patients still develop recurrence or metastasis within five years [ 3, 4] . Identifying novel biomarkers and developing more effective treatments are critical to overcoming these obstacles and improving patient survival.

RNA modification has emerged as a new frontier in the field of epigenetics and plays a crucial role in regulating gene expression and cell function. To date, more than 170 different types of RNA modifications have been identified, including N6-methyladenosine (m ^6^A), 5-methylcytosine (m ^5^C), N ^1^-methyladenosine (m ^1^A), and pseudouridine (Ψ) [5]. Among these modifications, m ^6^A is the most abundant and well-studied internal modification in eukaryotic RNA and is widely present in messenger RNA (mRNA), long non-coding RNA (lncRNA), microRNA (miRNA), and circular RNA (circRNA) [ 6– 8] . m ^6^A modification is dynamically regulated by a specific set of enzymes, including methyltransferases (“writers”), demethylases (“erasers”), and m ^6^A-binding proteins (“readers”) [9]. The coordinated action of these enzymes determines the m ^6^A landscape in cells and affects various aspects of RNA metabolism, including splicing, nuclear export, stability, translation, and degradation [10]. Abnormal m ^6^A modification is associated with the occurrence and development of cancer. Dysregulation of m ^6^A regulators can lead to abnormal gene expression, promoting tumorigenesis, proliferation, invasion, metastasis, and therapy resistance [ 11– 13] . For example, the m ^6^A writer METTL3 has been reported to be overexpressed in multiple cancers, including breast cancer, and is often associated with poor prognosis. METTL3-mediated m ^6^A modification can increase the stability and translation of oncogenic mRNAs, thus promoting the growth and survival of cancer cells [14]. Conversely, the downregulation of m ^6^A “erasers” (such as FTO and ALKBH5) can lead to increased m ^6^A levels and altered gene expression, which may also contribute to cancer development [ 15– 17] . For example, in TNBC, FTO overexpression leads to a significant reduction in m ^6^A modification levels of pri-miR-17, thereby inducing depletion of ZBTB4 expression and inhibiting the proliferation, migration, and invasion of TNBC cells [18]. Methyl-binding proteins can recognize and bind to RNA containing m ^6^A modifications, mediating the biological functions of m6A modifications. Common binding proteins include those in the YTHDF family (YTHDF1, YTHDF2, and YTHDF3), the YTHDC family (YTHDC1 and YTHDC2), and the IGF2BP family (IGF2BP1, IGF2BP2, and IGF2BP3) [ 19, 20] . The YTHDF family functions mainly in the cytoplasm. YTHDF1 can promote mRNA translation, YTHDF2 can accelerate mRNA degradation, and YTHDF3 can cooperate with YTHDF1 and YTHDF2. YTHDC1 is located mainly in the nucleus and participates in processes such as mRNA splicing and nuclear export [ 21, 22] . The IGF2BP family can recognize and bind to m ^6^A-modified mRNAs, increasing their stability and promoting their translation process [ 20, 23] . m ^6^A modification is a key regulator of breast cancer gene expression. It can not only affect the fate of mRNAs encoding key oncogenes in breast cancer development but also participate in the important modification of lncRNAs, thereby regulating important pathological processes such as the tumor cell cycle, apoptosis, angiogenesis, and metastasis [ 24, 25] .

LncRNAs are a class of non-protein-coding RNAs longer than 200 nucleotides [26]. They are dysregulated in breast cancer and can function as oncogenes or tumor suppressor genes through multiple mechanisms [ 27, 28] . LncRNAs can interact with DNA, RNA, or proteins to regulate gene expression at the transcriptional, post-transcriptional, or epigenetic level. In breast cancer, several lncRNAs (such as HOTAIR and MALAT1) have been shown to promote tumor growth, invasion, and metastasis, whereas other lncRNAs (such as MEG3) can inhibit the development of breast cancer [ 29– 31] .

Recent studies have revealed the complex interactions between m ^6^A modifications and lncRNAs in breast cancer. lncRNAs can regulate the expression and activity of m6A regulators, and m ^6^A-modified lncRNAs, in turn, can affect the functions of other RNAs or proteins in cells [32]. This m ^6^A-lncRNA axis may represent a novel regulatory mechanism in breast cancer. Understanding its molecular basis can provide new insights into the pathogenesis of breast cancer and potential therapeutic targets.

## The Molecular Mechanisms of the m ^6^A-lncRNA Axis

There is a close relationship between m ^6^A modification and lncRNAs. m ^6^A modification can affect the expression and function of lncRNAs, and lncRNAs can also regulate the activity of m ^6^A modification-related proteins. The m ^6^A-lncRNA axis formed by these two molecules plays a crucial role in tumors. Therefore, in-depth research on the molecular mechanism of the m ^6^A-lncRNA axis in tumors is highly important for revealing the pathogenesis of tumors and identifying potential therapeutic targets.

### Regulation of lncRNAs by m ^6^A modification

m ^6^A modifications play crucial roles in the processing and transportation of lncRNAs. Research has shown that METTL3-mediated m6A modification can affect the splicing process of lncRNAs. The m ^6^A modification of a specific lncRNA by METTL3 can alter its interaction with splicing factors, thus regulating the splicing pattern of the lncRNA and generating different splicing isoforms. These isoforms may have different functions, which in turn affect the biological behavior of tumor cells [ 33– 35] . In addition, m ^6^A modification is involved in regulating the transportation of lncRNAs from the nucleus to the cytoplasm. In hepatocellular carcinoma cells, METTL3 promotes the interaction between the lncRNA XIST and Exportin 5 through m ^6^A modification of XIST, thereby enhancing the transport of XIST from the nucleus to the cytoplasm and affecting the proliferation and metastasis ability of hepatocellular carcinoma cells. This process indicates that m6A modification can regulate the biological functions of lncRNAs in different cellular regions by altering their subcellular localization [ 36, 37] .

m ^6^A modification has a significant effect on the stability of lncRNAs, and this regulatory effect is achieved mainly through m ^6^A-binding proteins. YTHDF2, an important recognition protein for m ^6^A modification, can specifically bind to lncRNAs containing m ^6^A modifications, recruit relevant exonucleases, and promote the degradation of lncRNAs, thereby reducing their stability [ 38, 39] . In breast cancer cells, YTHDF2 can recognize and bind to the m ^6^A-modified lncRNA HOTAIR, promoting its degradation and thus inhibiting its proliferation and metastasis. Conversely, m ^6^A-binding proteins such as IGF2BP1-3 can bind to m ^6^A-modified lncRNAs, protecting them from nuclease degradation and increasing their stability [ 40, 41] . In lung cancer cells, IGF2BP2 binds to the m ^6^A-modified lncRNA MALAT1 to maintain its stability, thereby promoting the growth and metastasis of lung cancer cells [ 42, 43] . These findings indicate that the regulation of lncRNA stability by m ^6^A modification is complex. Different m ^6^A-binding proteins may have opposite effects on the stability of lncRNAs through different mechanisms, thus precisely regulating the expression level and biological functions of lncRNAs.

m ^6^A modification affects the interaction between lncRNAs and other molecules. m ^6^A modification can change the secondary and tertiary structures of lncRNAs, thereby influencing their interactions with other molecules, such as proteins, DNA, and RNA, and thus regulating their functions [ 44, 45] . In terms of interactions between proteins and lncRNAs, m ^6^A modification can enhance or weaken the ability of lncRNAs to bind to specific proteins. In colorectal cancer, the m ^6^A-modified lncRNA UCA1 can bind to hnRNPA2B1 to promote the proliferation and metastasis of colorectal cancer cells. The absence of m ^6^A modification affects the interaction between UCA1 and hnRNPA2B1, weakening its ability to promote tumor development [46]. In terms of the interaction between DNA and lncRNAs, m ^6^A modification can affect the binding affinity between lncRNAs and DNA. Some studies have shown that certain m ^6^A-modified lncRNAs can bind to specific DNA regions, recruiting chromatin-modifying complexes to regulate gene expression [ 47– 49] . In addition, m ^6^A modification can affect the interaction between lncRNAs and other RNA molecules. For example, m ^6^A-modified lncRNAs can interact with miRNAs to regulate the inhibitory effect of miRNAs on their target mRNAs, thus participating in the gene expression regulatory network [ 45, 50] . These results indicate that m ^6^A modification plays an important regulatory role in the occurrence and development of tumors by affecting the interaction between lncRNAs and other molecules.

### Regulation of m ^6^A modification by lncRNAs

LncRNAs can also regulate the expression and activity of m ^6^A modification-related proteins through multiple means, thereby affecting the m ^6^A modification level [51]. lncRNAs can affect the expression of m ^6^A modification-related proteins through transcriptional regulation, ceRNA mechanisms, etc. In colorectal cancer, the lncRNA SNHG1 can interact with miR-149-5p to upregulate the expression of METTL3, increase the m ^6^A modification level, and promote the proliferation and metastasis of colorectal cancer cells [ 52, 53] . In addition, lncRNAs can directly regulate transcription levels by binding to the promoter regions of genes encoding m ^6^A modification-related proteins [54]. LncRNAs can interact with m ^6^A modification-related proteins to affect their activity. In liver cancer, the lncRNA HULC can bind to METTL3, enhancing the methyltransferase activity of METTL3 and promoting the occurrence of m ^6^A modification [55]. In breast cancer, the lncRNA GAS5 can bind to FTO, inhibit its demethylase activity, increase the m ^6^A modification level, and affect the proliferation and apoptosis of breast cancer cells [56].

LncRNAs may also be involved in the selection and determination of m ^6^A modification sites. It can bind to RNA precursors, changing the local RNA structure and affecting the recognition of modification sites by the methyltransferase complex. When a specific lncRNA binds to a certain mRNA precursor, it causes the mRNA precursor to form a specific secondary structure, guiding the methyltransferase complex to perform m ^6^A modification in a specific region. This regulation of modification sites can precisely control the metabolic fate of RNA, thus affecting the biological functions of cells [ 57, 58] . In addition, lncRNAs may indirectly affect m ^6^A modification sites by forming complexes with other RNA or protein molecules. During neuronal development, the lncRNA Dubr interacts with the m ^6^A-binding protein YTHDF1/3 complex through its m ^6^A motif. This interaction protects YTHDF1/3 from degradation via the proteasome pathway and plays a crucial role in neuronal development [45]. In prostate cancer, the expression of non-coding RNA (FTO-IT1) located in the intron region of the *FTO* gene is significantly increased. Elevated FTO-IT1 inhibits the methyltransferase complex composed of METTL3/METTL14/WTAP/RBM1, reduces m ^6^A modifications on mRNAs and suppresses the stability and expression of p53 target gene mRNAs. The therapeutic deletion of FTO-IT1 restores the mRNA m ^6^A level in mice and the expression of p53 target genes and inhibits the growth of prostate cancer cells [59]. These complexes can change the localization and distribution of RNA molecules in cells, making it easier or more difficult for the methyltransferase complex to access certain potential modification sites, thereby regulating m ^6^A modification sites [60].

LncRNAs play important roles in maintaining the dynamic balance of m ^6^A modifications in cells to meet different physiological and pathological requirements [61]. When cells are stimulated by the outside world, the expressions of lncRNAs change, regulating the activity and expression levels of m ^6^A modification-related enzymes and causing corresponding changes in the m ^6^A modification level [ 50, 60] . When cells are under stress, the expression of certain lncRNAs is upregulated. It inhibits the activity of FTO, reduces the removal of m ^6^A modification, and promotes the expression of METTL3 to increase m ^6^A modification, thereby increasing the overall m ^6^A modification level in cells to cope with the stress environment [ 59, 62] . Moreover, lncRNAs can also maintain the dynamic balance of m ^6^A modifications through a feedback-regulatory mechanism. When the m ^6^A modification level changes, it affects the expression of related lncRNAs, and these lncRNAs in turn regulate the activity and function of m ^6^A modification-related enzymes, forming a complex regulatory network [ 57, 63] .

The regulatory mechanism of m ^6^A-lncRNA modification is complex and diverse. In-depth research on this regulatory mechanism is helpful for further revealing the mystery of RNA epigenetic regulation and provides new ideas and potential targets for tumor treatment.

## The m ^6^A-lncRNA Axis Regulates the Biological Function of Breast Cancer Cells

Increasing evidence indicates that m ^6^A-modified lncRNAs function as major regulators in modulating the biological functions of breast cancer cells, such as proliferation, apoptosis, migration, invasion, metastasis and therapy resistance. Previous studies have shown that m ^6^A-lncRNAs modulate the biological function of breast cancer cells by targeting multiple signaling pathways and molecules. The specific mechanisms are listed in Table 1.

**Table:** **Table 1** Relationships among lncRNAs, m ^6^A and their targets in the proliferation, apoptosis, cell cycle, migration, invasion, metastasis, EMT, and drug resistance of breast cancer cells

LncRNA	Expression in breast cancer	Related-m ^6^A	Target	Biological function	Ref.
WFDC21P	↑	METTL3	METTL3/WFDC21P/miR-628/SMAD3	Promotes proliferation, promotes migration	[64]
LINC00958	↑	METTL3	METTL3	Promotes proliferation, inhibits apoptosis	[65]
UCA1	↑	METTL14	METTL14-miR-375-SOX12	Promotes proliferation, promotes migration, promotes invasion	[66]
LNC942	↑	METTL14	LNC942/METTL14/CXCR4 and CYP1B1 signalling axis	Promotes proliferation, inhibits apoptosis	[57]
RUNX1-IT1	↑	IGF2BP1	IGF2BP1/GPX4 axis	Promotes proliferation, promotes migration, promotes invasion	[67]
MIR210HG	↑	IGF2BP1	MYCN/IGF2BP1/MIR210HG axis	Promotes proliferation	[68]
GAS5	↓	FTO	FTO/GAS5/IGF2BP2/QKI	Promotes proliferation	[56]
HOTAIR	↑	YTHDC1	Methylated A783, YTHDC1	Promotes proliferation, promotes migration, promotes invasion	[31]
LINC00667	↑	KIAA1429	KIAA1429/m6A/LINC00667/miR-556-5p	Promotes proliferation, promotes migration, promotes invasion	[69]
LINC00657	↑	—	M2/miR-92b-3p/TGF-β	Promotes proliferation, inhibits apoptosis, promotes migration, promotes invasion	[70]
NEAT1	↑	VIRMA	VIRMA	Promotes proliferation, inhibits apoptosis	[71]
AC084125.2	↓	METTL14	METTL14	Promotes migration, promotes invasion	[ 32, 72]
FGF14-AS2	↓	YTHDF2	YTHDF2	Promotes migration	[73]
LINC00115	↑	ALKBH5	SETDB1/PLK3/HIF1α/ALKBH5	Promotes migration, promotes therapy resistance	[24]
OIP5-AS1	↑	METTL3	METTL3/miR-150-5p/CCND2	Promotes migration, promotes invasion	[74]
MALAT1	↑	WTAP	WTAP	Promotes migration, promotes invasion, promotes EMT	[75]
FGD5-AS1	↑	—	has-miR-362-3p	Promotes therapy resistance	[76]
DLGAP1-AS1	↑	WTAP	WTAP/DLGAP1-AS1/miR-299-3p	Promotes therapy resistance	[77]
LINC01559	↑	FTO	FTO/YTHDF2/miR-1343-3p	Promotes therapy resistance	[78]
AGAP2-AS1	↑	METTL3	METTL3/YTHDF2	Promotes therapy resistance	[79]
MALAT1	↑	METTL3	METTL3/MALAT1/E2F1/AGR2	Promotes therapy resistance	[80]
A1BG-AS1	↑	IGF2BP2	IGF2BP2/ABCB1	Promotes therapy resistance	[81]
KCNQ1OT1	↑	METTL3	METTL3	Promotes therapy resistance	[82]

### Role of the m ^6^A-lncRNA axis in proliferation

**Figure 1:**
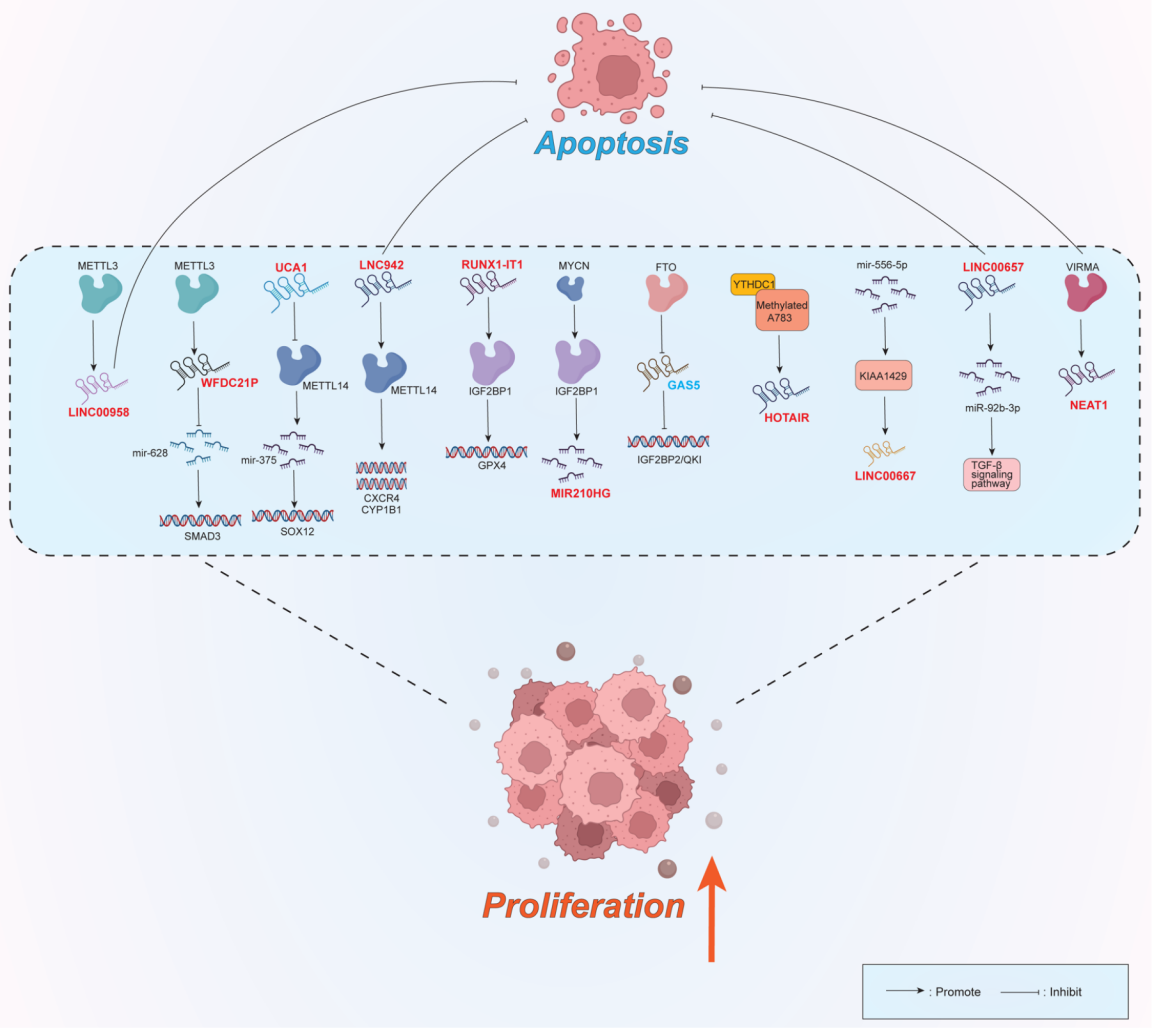
Figure 1 Several lncRNAs are regulated by various m ^6^A regulators and their signaling pathways to regulate the proliferation of breast cancer cells These genes included METTL3/miR-628/SMAD3, METTL14/miR-375/SOX12, METTL14/CXCR4/CYP1B1, IGF2BP1/GPX4, IGF2BP1/miR-210, FTO/IGF2BP2/QKI, YTHDC1, KIAA1429/miR-556-p, miR-92b-3p, and VIRMA. Oncogenic lncRNAs are denoted in red boldface; tumor-suppressive lncRNAs are denoted in blue boldface. Arrows denote activating/upregulatory effects; blunted lines denote inhibitory/downregulatory effects.

A series of studies have shown that lncRNAs modified by m ^6^A are closely related to the proliferation of breast cancer cells ( Figure 1). The writer METTL3 has been shown to affect breast cancer cell proliferation possibly by upregulating lncRNA WFDC21P expression [64]. Similarly, METTL3 specifically stimulates proliferation and inhibits the apoptosis of breast cancer cells by increasing the level of LINC00958 [65]. By targeting the METTL14-miR-375-SOX12 pathway, the knockdown of the lncRNA UCA1 inhibited breast cancer proliferation [66]. Sun *et al*. [57] reported that upregulated LINC00942 (LNC942) modulated the expression of CXCR4 and CYP1B1 through recruitment of the METTL14 protein, thus promoting the proliferation and inhibiting the apoptosis of breast cancer cells. In the “reader” family, IGFBP1/2 may explain the role of lncRNAs in breast cancer cell proliferation. Downregulation of the lncRNA RUNX1-IT1 was found to mediate dysregulation of glutathione peroxidase 4 (GPX4) by binding to the m ^6^A reader IGF2BP1, thereby reducing the proliferation of breast cancer cells and significantly increasing apoptosis [67]. Shi *et al*. [68] reported that the lncRNA MIR210HG, which is regulated by the m6A recognition protein IGF2BP1, can also induce the proliferation of breast cancer cells by regulating its encoded miR-210. In addition, the lncRNA GAS5, which is regulated by eraser (FTO), specifically inhibits the proliferation of breast cancer cells by targeting the FTO/GAS5/IGF2BP2/QKI pathway [56]. Another type of “reader” also plays an important role in the proliferation of breast cancer cells. For example, persistent methylation of a m ^6^A site (A783) on the lncRNA HOTAIR was recently shown to enable the m ^6^A reader YTHDC1 to interact with HOTAIR, thereby stimulating the proliferation of triple-negative breast cancer cells [40]. LINC00667, a highly m ^6^A-modified lncRNA, was found to be elevated in breast cancer cells and was induced by the overexpression of KIAA1429, thus promoting the proliferation of breast cancer cells [69]. The level of LINC00657 is positively correlated with the methylation level of m ^6^A, and its overexpression can significantly induce the proliferation of breast cancer cells and slow their apoptosis [70]. The upregulation of VIRMA, an m ^6^A methyltransferase-related protein, promoted the proliferation and inhibited the apoptosis of breast cancer cells by increasing the expression of the lncRNA NEAT1 [71].

### Role of the m ^6^A-lncRNA axis in migration, invasion, metastasis, and EMT

**Figure 2:**
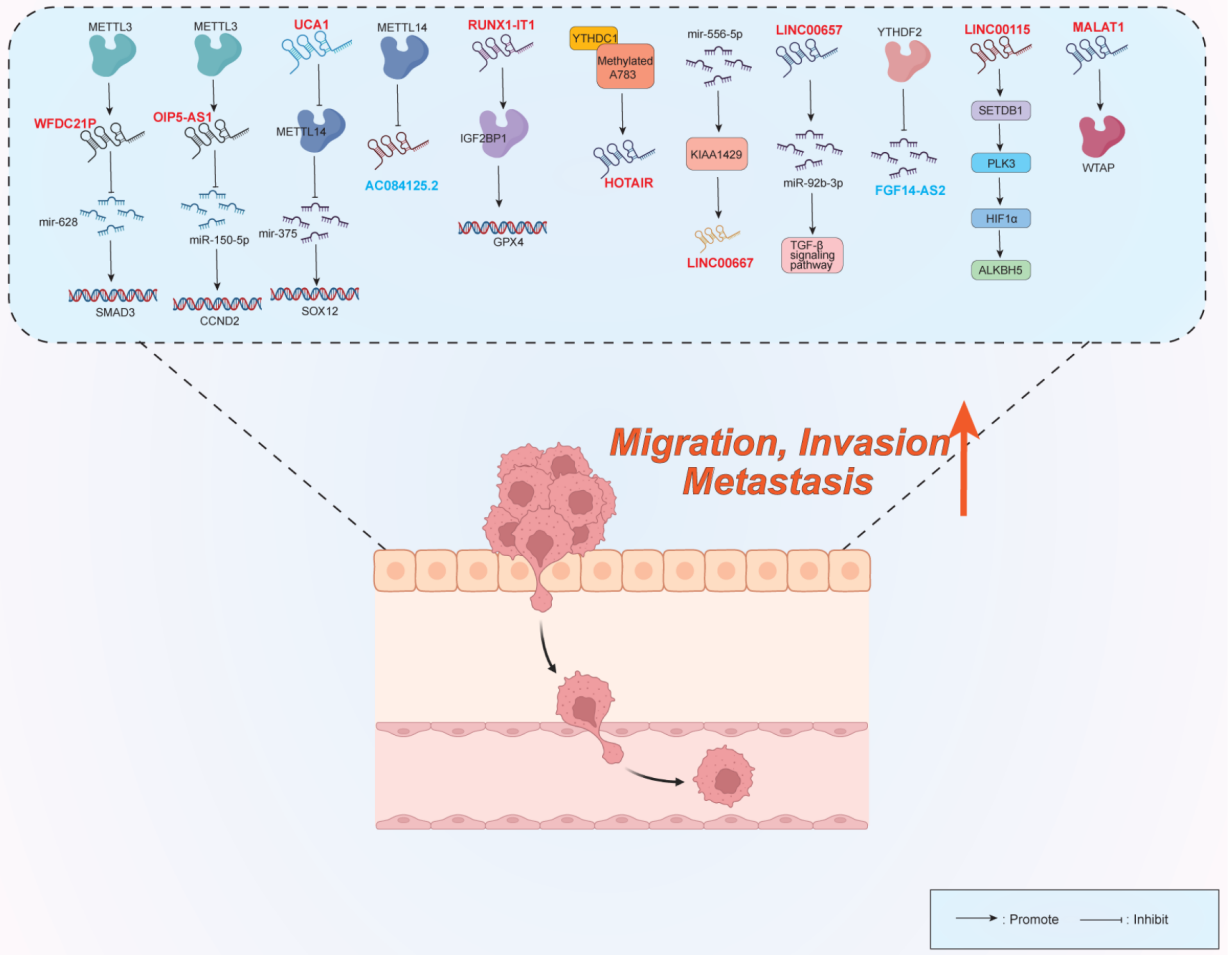
Figure 2 The function of the m ^6^A-lncRNA axis in cell migration, invasion, metastasis, and EMT Methylated A783/YTHDC1, METTL3/miR-628, KIAA1429/miR-556-5p, miR-92b-3p/TGF-β, METTL14/miR-375/SOX12, IGF2BP1/GPX4, miR METTL14 and other lncRNAs affected by m6A regulate the migration, invasion and metastasis of breast cancer cells. By targeting YTHDF2, the lncRNAs FGF14-AS2, LINC00115, OIP5-AS1, MALAT1, SETDB1/PLK3/HIF1α/ALKBH5, METTL3/miR-150-5p/CCND2, WTAP and other genes regulate the migration, invasion and metastasis of breast cancer cells. Oncogenic lncRNAs are denoted in red boldface; tumor-suppressive lncRNAs are denoted in blue boldface. Arrows denote activating/upregulatory effects; blunted lines denote inhibitory/downregulatory effects.

Recent studies have demonstrated that m ^6^A methylation influences the invasion and metastasis of breast cancer cells by regulating multiple long non-coding RNAs (lncRNAs), such as HOTAIR. The underlying molecular mechanisms hold great potential for identifying therapeutic targets for breast cancer treatment ( Figure 2). Sustained m ^6^A methylation at A783 was recently shown to regulate lncRNA HOTAIR overexpression, thereby increasing the aggressiveness of TNBC cells [40]. Knocking down the lncRNA MALAT1 can downregulate the expression of the WTAP protein in Writer cells and restrict the invasion and metastasis of TNBC cells, which is related to the inhibition of the EMT process [75]. METTL3 is a key factor in regulating lncRNA function through m ^6^A modification [83]. For example, overexpressed METTL3 affects the m ^6^A modification of lncRNA-WFDC21P, promoting the metastasis of breast cancer through the WFDC21P/miR-628/SMAD3 axis [64]. Wu *et al*. [74] reported that silenced lncRNAOIP5-AS1 activated the miR-150-5p/CCND2 axis by binding to METTL3, promoting the migration and invasion of breast cancer cells. Similarly, METTL14 is closely related to the m ^6^A modification of lncRNAs, and its overexpression inhibits the expression of the lncRNA AC084125.2, which hinders cell migration and invasion [ 32, 72] . LncRNAUCA1 upregulated the level of METTL14, further promoting the m ^6^A modification of miR-375 and the expression of SOX12 in breast cancer cells, promoting the migration and invasion of breast cancer [66]. The knockdown of the lncRNA RUNX1-IT1 inhibited breast cancer migration and invasion and caused its reduced binding to IGF2BP1, resulting in reduced stability of GPX4 [67]. MIR210HG has been shown to be an oncogene that may be positively correlated with breast cancer cell metastasis, and it could fulfil its biological function through miR-210 [68]. The lncRNA FGF14-AS2 inhibits the translation of RUNX2 by suppressing the assembly of the eIF4E/eIF4G complex and the phosphorylation of eIF4E, reducing the transcription of RANKL, a key regulatory factor of osteoclast differentiation, and inhibiting bone metastasis in breast cancer. However, LncRNAFGF14-AS2 is downregulated by RNA degradation mediated by YTHDF2 in an m ^6^A-dependent manner, resulting in poorer distant metastasis-free survival in patients with high expression of YTHDF2 and low expression of FGF14-AS2 [73]. Upregulated LINC00115 enhances breast cancer cell metastasis by actively targeting SETDB1 and PLK3 to reduce m6A methylation levels [24]. Ren *et al*. [69] reported that LINC00667 is an m ^6^A-modified lncRNA and that its high expression is associated with a poor prognosis in patients with breast cancer. LINC00667 positively regulates KIAA1429 by sponging miR-556-5p. KIAA1429 can also bind to the m ^6^A modification site of LINC00667, enhancing the stability of its mRNA. A KIAA1429/m6A/LINC00667/miR-556-5p feedback loop is formed. These findings suggest that targeting KiAA1429-induced LINC00667 expression provides potential possibilities for breast cancer treatment through an m ^6^A-dependent feedback loop. Additionally, LINC00657 is significantly upregulated in breast cancer-derived exosomes and is associated with increased m ^6^A methylation levels. LINC00657-overexpressing breast cancer cells activate the TGF-β signaling pathway by sequestering miR-92b-3p in macrophages, thereby inducing M2 polarization of macrophages. M2-polarized macrophages, in turn, promote the invasion and migration of breast cancer cells, forming a reciprocal regulatory loop [70].

### Regulatory effects of the m ^6^A-lncRNA axis on the therapeutic resistance of breast cancer

Chemotherapy occupies a central position in the treatment of breast cancer. Anthracycline and taxane drugs, which serve as the mainstays of breast cancer chemotherapy, provide a guarantee for the survival benefit of patients. Compared with nonanthracycline regimens, chemotherapy regimens containing anthracycline drugs significantly improve the disease-free survival (DFS) and overall survival (OS) of patients [84]. International multicenter studies have also shown that the combination of paclitaxel and anthracyclines for adjuvant chemotherapy of early breast cancer greatly reduces the risk of recurrence [85]. Platinum drugs are important therapeutic agents for TNBC and play crucial roles in the treatment of neoadjuvant, adjuvant, and advanced breast cancer. The CBCSG006 study revealed that in the neoadjuvant treatment stage of TNBC, the objective response rates (ORRs) of the gemcitabine combined with cisplatin (GP) regimen are 67.9% and 50.4% greater than those of the gemcitabine combined with paclitaxel (GT) regimen; the median overall survival (OS) time is 22.40 months and 18.53 months, respectively, and the GP regimen is significantly more effective than the GT regimen [86]. For metastatic triple-negative breast cancer, the results of the GAP study suggested that the median progression-free survival time of the nab-paclitaxel combined with cisplatin group was significantly greater than that of the gemcitabine combined with cisplatin group (9.8 months and 7.4 months, respectively) [87]. In the treatment of advanced breast cancer, a meta-analysis that pooled data from multiple clinical studies revealed that platinum drugs significantly prolong the median progression-free survival of patients with advanced TNBC [88]. These studies indicate that preintervention chemotherapy can effectively control the progression of breast tumors and improve the overall treatment effect. Resistance to chemotherapy, targeted therapy or immunotherapy has hindered the development of cancer treatments [89]. Therapy resistance is a major challenge in the treatment of breast cancer, and its mechanism is complex [90].

The discovery and in-depth research of the m ^6^A-lncRNA axis provide a new direction for deciphering the mechanism of therapy resistance in breast cancer and are expected to lead to breakthrough progress in the treatment of this disease. Xu *et al*. [76] reported that the competitive binding of the m6A-modified lncRNAs FGD5-AS1 and hsa-miR-362-3p affects the sensitivity of breast cancer to cisplatin. Huang *et al*. [77] reported that lncRNA DGAP1-AS1 modified by m ^6^A promoted therapy resistance in breast cancer cells through the feedback regulatory mechanism of WTAP/DLGAP1-AS1/miR-299-3p. Specifically, the m ^6^A methyltransferase WTAP mediates DLGAP1-AS1 upregulation. DLGAP1-AS1 binds to miR-299-3p through its 3′-UTR, and in turn, miR-299-3p targets the WTAP 3′-UTR. In addition, FTO inhibited the enrichment of m ^6^A-modified LINC01559 mRNA in an m ^6^A-YTHDF2-dependent manner and promoted the expression of LINC01559 in breast cancer patients, which might be related to docetaxel resistance [78]. The overexpression of the lncRNA AGAP2-AS1 reduces the sensitivity of breast cancer cells to trastuzumab through METTL3/YthDF2-mediated m ^6^A methylation [79]. Metastasis-associated lung adenoma transcription-1 (MALAT1) is a highly conserved lncRNA that mediates the E2F1/AGR2 axis through m ^6^A modification of METTL3, increasing doxorubicin resistance in breast cancer cells [80]. LINC00115 reduces m6A modification by activating HIF1α signalling via the SETDB1/PLK3 and ALKBH5/YTHDF2 complexes, thereby promoting the resistance of breast cancer cells to chemotherapy [24]. The knockdown of lncRNA A1BG antisense RNA1 (A1BG-AS1) increased the expression of ATP-binding box subfamily B member 1 (ABCB1, also known as MDR1) by recruiting the m ^6^A reader IGF2BP2, which negatively affects the activity of drug-resistant breast cancer cells [81]. Zhou *et al*. [82] reported that silencing METTL3 induced apoptosis by increasing the expression of m6A-dependent lncKCNQ1OT1 and then promoted doxorubicin (DOX) resistance in breast cancer cells. Furthermore, Wan *et al*. [91] used the Arraystar human m ^6^A mRNA & lncRNA epigenetic transcriptome microarray and reported a positive correlation between METTL3/IGF2BP3 expression and PD-L1 expression in HER2-positive and TNBC cancer tissues, leading to adaptive immune resistance and ultimately promoting the occurrence and development of breast cancer. This process likely occurs via lncRNA expression regulation. In pancreatic cancer, METTL3 positively modulates the lncRNA MALAT1 to upregulate PD-L1 in pancreatic cancer cells [92]. In non-small cell lung cancer, METTL3 promotes the YTHDF2-mediated degradation of LINC02418, a negative regulator of PD-L1 [93]. Additionally, numerous m ^6^A-associated lncRNAs are linked to the expression of immune checkpoint molecules, including CTLA-4, LAG-3, TIM-3 and TIGIT, across breast, esophageal, thyroid and other cancers. While the mechanisms underlying m6A-lncRNA regulation of these checkpoints remain undefined, established PD-L1 regulatory paradigms may inform future investigations.

Therapeutic resistance may be associated with dynamic reprogramming of the m6A-lncRNA axis under therapeutic pressure [94]. Mechanistically, treatment-induced stress elicits oscillatory expression of m6A modifiers, orchestrating lncRNA expression to drive resistance. For example, in docetaxel-resistant breast cancer models, Zhou *et al*. [82] demonstrated that METTL3 upregulation in resistant cells exceeds that in normal breast cells, governing downstream lncKCNQ1OT1 to promote drug resistance. Concurrently, WTAP-mediated upregulation of the lncRNA DLGAP1-AS1 contributes to doxorubicin resistance phenotypes [77]. In HER2-targeted therapy resistance, METTL14 downregulation diminishes m ^6^A methylation in patient-derived xenografts and organoids, sustaining trastuzumab resistance [95]. Moreover, treatment pressure remodels m ^6^A deposition across subcellular compartments, driving spatiotemporal reorganization of m ^6^A-lncRNA networks to facilitate resistance establishment [ 96, 97] .

## The Role of the m ^6^A-lncRNA Axis in the Early Diagnosis and Prognosis of Breast Cancer and Its Potential Clinical Application

Recent studies on the role of the m ^6^A-lncRNA axis in breast cancer may help to discover biomarkers that can be used for the early diagnosis and prognosis of breast cancer. Lv *et al*. [98] screened six m ^6^A-related lncRNAs (Z68871.1, AL122010.1, OTUD6B-AS1, AC090948.3, AL138724.1, and EGOT) from The Cancer Genome Atlas (TCGA) database and established a prognostic risk model for predicting OS in breast cancer patients. According to the model, patients can be effectively divided into patients with good and poor prognoses. Similarly, Yao *et al*. [99] analyzed the expression profiles of lncRNAs, mRNAs and miRNAs in breast cancer through the TCGA database for weighted gene coexpression network analysis and used standard Kaplan-Meier univariate curves to analyze the correlation between clinical information. Five lncRNAs (AL117190.1, COL4A2-AS1, LINC00184, MEG3, and MIR22HG) were identified as key factors for the prognosis of breast cancer patients. The progress of immunotherapy offers hope for the treatment of breast cancer. Zhang *et al*. [100] proposed that the m ^6^A-related lncRNA model can be used as a prognostic marker and immunotherapy target for breast cancer treatment. By analysing the coexpression of lncRNAs related to m ^6^A, breast cancer patients were divided into different subgroups. Four molecular subtypes were identified through consistent clustering, and the prognostic characteristics of lncRNAs related to m ^6^A were generated. Twenty-one lncRNAs related to m ^6^A can be used to construct a lncRNA model related to m ^6^A (m ^6^A-lnCRM). Survival analysis and receiver operating characteristic (ROC) curve analysis further confirmed the prognostic value and predictive performance of the model. A total of 937 immune-related lncRNAs were identified through coexpression analysis of immune-related genes, with the aim of identifying immune-related lncRNA signals to improve the prognostic prediction of patients with breast cancer [101]. Fifteen candidate immune-related lncRNAs were significantly correlated with OS. Eight lncRNAs (OTUD6B-AS1, AL122010.1, AC136475.2, AL161646.1, AC245297.3, LINC00578, LINC01871, and AP000442.2) were selected to establish a risk prediction model. Multivariate Cox regression analysis indicated that this model was an independent and reliable indicator for the prognosis of patients with breast cancer in the training set. In addition, lncRNAAL122010.1, identified as a biomarker for the diagnosis and prognosis of breast cancer patients, is associated with breast cancer stem cells [102], autophagy [103], and immunity [101]. Cui *et al*. [104] identified seven lncRNA models related to m ^6^A (LRRC8C-DT, COL4A2-AS1, AP005131.2, AL138789.1, AC012213.3, U73166.1, and TFAP2A-AS1) from the TCGA database. This model accurately predicts the prognosis of breast cancer patients and has the potential to improve the TNM staging system. Zhu *et al*. [105] discovered that the hypoxia-induced lncRNA KB-1980E6.3 accelerated the self-renewal capacity and tumor growth of breast cancer stem cells through interacting with IGF2BP1 and stabilizing c-Myc mRNA. Consequently, targeting the lncRNA KB-1980E6.3/IGF2BP1/c-Myc signaling axis may pave a new therapeutic pathway for treating hypoxic breast cancers that are resistant to traditional therapies. Hu *et al*. [106] developed a fluorescence resonance (FRET) nanosensor based on single quantum dots (QDs), which showed high sensitivity at the single-cell level. The nanosensor, which can accurately track the level of m6A modification at specific loci of lncRNAs in cells, deeply analyzes the specific expression profile of m ^6^A in breast cancer tissues and neighboring healthy tissues, providing strong support for breast cancer treatment.

## Conclusions and Prospects

LncRNAs and m ^6^A represent crucial and intricate regulatory mechanisms in the field of epigenetics and play key roles in the occurrence and progression of breast cancer, including cell proliferation, apoptosis, migration, metastasis, invasion and therapeutic resistance. Investigations into the m ^6^A-lncRNA axis offer valuable insights and innovative mechanisms for elucidating the onset and progression of breast cancer and direct drug development for breast cancer in the future. However, the current understanding of the role of the m ^6^A-lncRNA axis in breast cancer remains in the exploratory phase. Consequently, there is an urgent need to establish novel approaches for investigating and elucidating the multifaceted functions of the lncRNA-m ^6^A axis. The primary challenge in breast cancer treatment stems from the heterogeneity of molecular features and their dynamic regulation across temporal and spatial dimensions. This also represents a promising direction for future research. Further investigations are warranted to elucidate whether and how the m ^6^A-lncRNA axis contributes to the regulation of this process.

In this review, we systematically summarized and discussed the mutual regulatory mechanisms between lncRNAs and m ^6^A, which may serve as promising potential therapeutic targets and predictive biomarkers for breast cancer. Within the cell, there is a highly crowded environment of biomolecules and an intricate network of regulatory interactions.

Here, we not only explored the correlation between lncRNAs and m ^6^A but also revealed that the m ^6^A-lncRNA axis is closely related to breast cancer. LncRNAs regulate the proliferation, apoptosis, therapeutic resistance, migration, invasion, metastasis and EMT of breast cancer cells by regulating m ^6^A methylation. In addition, lncRNAs modified by m ^6^A are expected to be new prognostic targets and biomarkers that have important clinical application potential. Notably, the same molecule can play dual oncogenic or tumor-suppressive roles in distinct breast cancer contexts. Take YTHDF2 as an illustrative example. In TNBC, YTHDF2 facilitates the degradation of FGF14-AS2, thereby promoting the osteolytic metastasis of breast cancer cells [73]. Conversely, YTHDF2 has also been shown to suppress cell proliferation by degrading HOTAIR [38]. These apparently conflicting effects may be attributed to “context-dependent” mechanisms. On the one hand, breast cancers with different molecular subtypes present unique gene expression profiles and activated signaling pathways, leading to variations in YTHDF2-targeted RNA substrates and their downstream functional consequences. On the other hand, factors within the tumor microenvironment, such as hypoxia, inflammatory responses, or interactions with stromal and immune cells, can dynamically modulate YTHDF2 function. Therefore, when investigating the role of the m ^6^A-lncRNA regulatory network in breast cancer, it is essential to adopt a research paradigm that is multidimensional, dynamic, and sensitive to environmental cues.

Other noncoding RNAs are also modified by m ^6^A, thereby regulating the onset and progression of breast cancer. For example, the interactions between miRNAs and a variety of m ^6^A regulators play crucial roles in this process [107]. MiR-483p downregulates the m ^6^A methylation level of p21 by binding to the 3′-UTR of *METTL3*, inhibiting breast cancer cell proliferation [108]. Moreover, METTL3 was shown to modulate m ^6^A levels and docetaxel resistance in breast cancer via the regulation of LINC00662 and miR-186-5p expression [109]. Circular RNAs (circRNAs) and P-element-induced wimpy testis (PIWI)-interacting RNAs (piRNAs), a novel class of noncoding RNAs, exhibit comparable functional mechanisms. CircMETTL3 functions as a competitive endogenous RNA (ceRNA) for miR-34c-3p, increases the expression level of METTL3, and consequently enhances breast cancer cell proliferation, invasion and metastatic potential [110]. Lv *et al*. [111] demonstrated that circBACH2 activates the MAPK signaling pathway by sequestering hsa-miR-944, resulting in increased HNRNPC expression and breast cancer cell proliferation. Furthermore, piRNA-31106 overexpression has been found to promote breast cancer progression through METTL3-mediated m ^6^A methylation [112]. Therefore, future studies should focus on further elucidating the regulatory mechanisms between the m ^6^A-lncRNA axis and other epigenetic regulatory pathways, thereby gaining a deeper understanding of its critical role in breast cancer.
